# Psychosocial Profile of Juvenile and Adult Offenders Who Acknowledge Having Committed Child-to-Parent Violence

**DOI:** 10.3390/ijerph19010601

**Published:** 2022-01-05

**Authors:** Ana M. Martín, Leticia De la Fuente, Antonia Hernández, Flor Zaldívar, Elena Ortega-Campos, Juan García-García

**Affiliations:** 1 Department of Cognitive, Social and Organizational Psychology, Universidad de La Laguna, 38296 La Laguna, Spain; ammartin@ull.edu.es; 2 Health Research Center, Universidad de Almería (CEINSA/UAL), 04120 Almería, Spain; lfuente@ual.es (L.D.l.F.); flor@ual.es (F.Z.); elenaortega@ual.es (E.O.-C.); 3 Department of Psychology, Universidad de Almería, 04120 Almería, Spain; 4 Fundación Canaria de Juventud Ideo, 38005 Santa Cruz de Tenerife, Spain; hreyesto@hotmail.com

**Keywords:** child-to-parent violence, exposure to violence, interpersonal problem-solving skills, juvenile and adult justice

## Abstract

The main objective of this study was to establish the psychosocial profile of adolescents and adults who have admitted to committing child-to-parent violence (CPV) and were serving a judicial sanction or prison sentence, respectively. Two groups of participants took part in this study. The first group was made up of 89 male youths who were serving judicial sanctions, and the second group was made up of 70 men serving a prison sentence. A cross-sectional retrospective design with concurrent measurements was used in this study. Group differences in the exposure-to-violence variables were conducted. Automatic regression models were used to estimate a self-reported CPV. In relation to the variables of indirect exposure to violence, statistically significant differences between those who admitted having committed CPV and those who did not, irrespective of being adults or adolescents, were found for seeing violence in class and at home but not for seeing violence on the street or on television. Regarding the variables related to experiencing violence, the results showed statistically significant differences in experiencing violence at home but not in class or on the street. The best predictive model of CPV includes some of the dimensions of self-concept, specifically academic and family self-concept, as well as the avoidant and rational problem-solving styles and the negative orientation toward problems. The results have shown the existence of a CPV offender profile that is common to minors and adults.

## 1. Introduction

In many countries, including Spain, child-to-parent violence (CPV) is the type of intrafamily violence that has taken the longest to come to light. There is general agreement that CPV in this country has taken a turn for the worse since late 2003, but there is no such agreement on why this is so [1]. We find a possible answer in the 2003 reform of Penal Code articles 153 (on abuse) and especially article 173.2 (on habitual abuse) when the new Organic Law 11/2003 came into force. This modified legislation referred to all types of intrafamily violence and constituted “a decisive element in the marked increase in cases” of this type since they were recognized with more severity; cases that previously were considered only misdemeanors became redefined as crimes. As a result, there was a “perpetual increase in the number of complaints filed by victims” who, in many cases, were seeking civil measures for protection, not only criminal measures [2] (p. 580). This assessment, given by the Attorney General at that time, refers to victims of domestic violence in general, but given the data provided on the aggressor’s age and their relationship to the victim, we find indications of CPV prevalence in particular. The following year, the media published a great number of articles calling attention to this increase. Emphasis was on the young age of certain perpetrators, and all kinds of explanations for CPV were being put forward, but there were no allusions to the legislative reform that had made this violence visible to the public eye [3].

The Attorney General’s Office did not explicitly refer to “attention that should be given to the physical and psychological abuse of offspring against parents” until the 2006 Annual Report [4] (p. 159). It was the 2007 report where, for the first time, there was a section entitled “Family abuse of parents by their offspring and its problematic treatment in criminal law”, in addition to the statement that “parental abuse, together with sibling abuse, constitutes the most common form of non-gender-based domestic violence” [5] (p. 641). In the following years, these annual reports repeatedly call out the increase in the number of cases, and that the effort of justice and reformation entities seemed insufficient to address this problem [6] (p. 413), going so far as to state that “it is disheartening to observe how this modality of crime rises year after year” [7] (p. 938).

The fact that CPV has not been visible in society does not mean that it did not exist or that it had not been denounced from the most ancient times. Law 195 of the Code of Hammurabi stated, in the year 1750 BC, that if a son hits his father, his hands should be cut off. Even then, it was considered “something unnatural that deserved severe punishment”, unlike child abuse and intimate partner violence, which took longer to become the object of social and legal reproach [1] (p. 20). Among the peculiarities of CPV that might explain why it has only recently become the focus of legal, media, and scientific attention, the following stand out: (1) “the profile of the perpetrators does not fall within the usual context of poverty or marginality where juvenile delinquency appears”, (2) “the perpetrator’s affective connection with the victims”, (3) “the need to duly protect [the victims] and also protect [the aggressor] when removal from the family environment is imposed”, and (4) the fact that “specific interventions are being called for … that are not always among the resources available in certain territories” [8] (p. 1050).

Regarding the offender’s profile, since 2005 [3], the media has pointed to the fact that many of the adolescents reported for CPV were part of apparently normal families; they had a medium to high socioeconomic level, with no previous associated psychopathologies, were not violent outside the home, and had not committed other types of crimes [9,10]. This “new” delinquency profile was opposed to the “traditional” profile, where CPV forms part of a larger problem, such as a serious psychopathology, use of toxic substances, or a pattern of response to neglect or previous violence (“defensive” violence) [9]. This “new” profile refers to adolescents who manifest defiant behaviors of disobedience (“offensive” violence) and is considered the result of inconsistent parenting standards and the lack of limits from parents who are trying to avoid any type of frustration for their offspring [11,12,13]. In Circular 1/2010 [10], the Attorney General’s Office adopted this profile and this underlying causal explanation in the juvenile justice system’s treatment of adolescents who abuse their parents. However, scientific research has shown that the risk factors associated with CPV are diverse and have to do with characteristics of the aggressors, the victims, and their families, all of which indicate why some adolescents are more likely to perpetrate CPV than others [14,15].

One of the most consistent results of research in this field is that adolescents who assault their parents have more often been victims of abuse in the family or have witnessed violent behavior between their parents than those who do not [16,17,18,19,20,21,22,23,24,25,26,27]. Any kind of exposure to intrafamily violence can affect the normal development of offspring [28], such that those who have experienced or observed such violence are more likely to develop different cognitive, emotional, and/or behavior problems in childhood and to manifest violence and antisocial behaviors in adolescence [29,30,31] and adulthood [32]. These consequences apply to both direct and indirect victimization since both of these lead to the belief that the use of violence is justified as a form of conflict resolution of any kind [17,33,34,35,36,37]. Previous exposure to violence, as well as the belief that the use of violence is justified, would be able to explain the co-occurrence of CPV and intimate partner violence in adolescents [38,39] and their continued practice over time [40].

This type of data has led to the bi-directionality of violence hypothesis [27,41] to be useful in explaining CPV. This hypothesis suggests that exposure to violence in the family, whether directly as a victim or indirectly as a witness, always precedes CPV [20,26]. In one of the first longitudinal studies on this topic, Brezina [41] found that victimization suffered by male adolescents and its frequency predicted aggression toward parents one and a half years later. The verification that this relationship is reciprocal served to show that parents’ aggression towards offspring promoted the offspring’s assault of their parents. In this way, the aggression of offspring towards their parents is an indicator of aggressive parental behavior towards them. In Spain, several studies [21,42] have contributed data similar to those of Brezina [41], thereby supporting the bi-directionality of violence hypothesis. In both studies, aggressive family interactions were reciprocal, given that parents who exercised violence against their offspring became victims of violence from their offspring more often than those who did not. The percentage of CPV offenders who report having been victims of their parents ranges between 60% for prosecuted adolescents [43] and 38% in community populations [44].

The results of previous studies also concur with the theory of the intergenerational transmission of violence, according to which the offspring of violent parents have a greater probability of being violent when they reach adulthood, even when they have not been direct victims of such violence [45]. This theory has been used to explain the relationship between intimate partner violence in the offspring’s family of origin and violence in later intimate relationships [45], as well as between having been a victim of child abuse and abusing offspring as an adult [46].

Low empathy is another characteristic associated with the offender’s profile in CPV cases [47,48,49]. Lack of empathy has been linked to aggressive and antisocial behavior in general [50,51], but its relationship to criminal behavior is moderate and is limited to the cognitive dimension [52]. Regarding CPV, several studies [48,49,53] found statistically significant differences in the levels of empathy that supervisors of judicial sanctions attributed to adolescents sentenced for CPV compared to those sentenced for other crimes. Using self-report questionnaires, Castañeda et al. [47] found that a group of adolescents who were serving judicial sanctions for CPV obtained higher scores on the MACI Social Insensitivity scale in comparison to a population of high school students, although the difference was much less than differences found in the Expressed Concerns scales on Childhood Abuse and Family Discord. In the study by Contreras et al. [54], empathy was not related to any of the elements of the CPV model that the authors tested.

Similarly, low self-esteem has been related to aggressive behavior in general [55], although, in the case of CPV, this association is only moderate when dealing with normal populations [17,18,22,56,57,58,59]. In samples of persons serving judicial sanctions, low empathy is attributed to CPV offenders in the reports by their supervisors or in their files [48,49,51]. However, when instruments of measure are used, such as self-report questionnaires, the association does not reach statistical significance [15,43,60,61,62].

At this point, it is important to note that results obtained from judicial files, while valuable, ought to be considered with caution for several reasons. First, the information that the researcher is using was collected for other purposes and is reflected in the files in qualitative terms, or in the best case, dichotomously (yes/no). Second, the professionals who write up each file may have different professional training (psychologists, educators, social workers), and the assessments they make are clinical judgments usually based on semi-structured interviews and not on psychometric instruments, as is the case with self-esteem. Third, at the time the evaluation is carried out, the assessors know the crime that the adolescent is accused of or for which s/he is serving sentence, so their expectations may have an important effect on their evaluation [63]. In addition to problems stemming from the type of measurement used, the lack of consistency in results pertaining to self-esteem may be due to its dependence on external events, especially in a developmental transition period, such as adolescence. For this reason, Hernández et al. [64] considered that self-concept, measured with self-reports, was a more appropriate construct to be studied in relation to CPV than self-esteem, given its greater stability over time and the possibility of differentiating between several facets of the adolescent’s life (social, emotional, family, academic, and physical). Their results showed that there were statistically significant differences between adolescents with and without judicial sanctions, but only in relation to the family dimension of self-concept. The normal population of adolescents scored the highest, and the lowest scorers were for those serving judicial sanctions for CPV-related crimes; those sentenced for other crimes fell between the two groups.

Interpersonal problem-solving skills have also been related to CPV, although this link has received less empirical study. These skills underlie the cognitive process by which people handle conflictive interactions in their daily life [65]. Nock and Kazdin [66] found that adolescents from clinical samples who received therapy for exercising CPV had difficulties adequately responding to certain problematic interpersonal situations. In the study by Contreras and Cano [60], adolescents under judicial sanctions showed significantly lower levels of field independence, as well as greater impulsivity, and were less able to notice and retain relevant information about social situations when compared to those not under judicial sanctions. In addition, these adolescents were differentiated both from those who had no judicial sanction and from those who had committed other crimes by showing less ability to anticipate and understand the consequences of social behaviors or to select the appropriate means for reaching their objectives.

These results are consistent with studies that have repeatedly confirmed how deficiencies in interpersonal problem-solving skills are related to personality disorders [67], as well as to aggressive behavior, drug use, and delinquency [68,69,70,71,72]. They also concur with studies where the ability to adequately solve interpersonal problems is shown to be a protective factor against aggressive and antisocial behaviors [69] and that the most effective intervention programs with offenders are cognitive-behavioral, focused on training in interpersonal problem-solving skills, and include self-control, creative thinking, critical reasoning, values training, and meta-cognition [17,73,74,75].

The profile of CPV offenders reflected in previous research also characterizes them as adolescents, although there are data indicating that between 75% and 91% of parricides, the most extreme form of CPV, are committed by adults [76]. In continued CPV, there is an escalation of violence as in other forms of abuse, so that the most serious behaviors are preceded and/or accompanied by milder and less censured forms [77]. In this regard, the Attorney General’s office, in its 2020 annual report, expressed concern not only that CPV had become a “criminal problem embedded in the social fabric”, but because it is not known what has become of those who battered their parents in adolescence and are now adults, probably with offspring of their own (p. 939).

The absence of data on CPV in the adult population could be the result of the social construction of the aggressor as an adolescent [78], associated with the traditional conception of adolescence as the most conflictive and difficult stage of development [79]. Similarly, since CPV falls under the legal category of domestic violence, and since the media uses this category almost exclusively for referring to intimate partner violence, it is assumed that adults serving a sentence for this crime have assaulted their spouse and not their parents [78].

The main objective of this study was to establish the psychosocial profile of adolescents and adults serving a prison sentence or a judicial sanction, respectively, who have admitted to having carried out CPV. For this purpose, several sequenced, interrelated objectives have been put forward. Firstly, we explore whether direct or indirect exposure to different forms of violence is a common pattern in both groups, such that the two groups may be considered equivalent in terms of this exposure. Secondly, we examine whether exposure to violence is a differential factor between those who admit to having practiced CPV and those who do not, whether they are adolescents or adults. Finally, a CPV predictive model is proposed based on dimensions of the psychological variables of empathy, self-concept, and problem orientation and problem-solving styles.

## 2. Method

### 2.1. Study Design

A cross-sectional retrospective design with concurrent measurements was used in this study.

### 2.2. Participants

Two groups of Spanish participants took part in this study that was carried out in the Canary Islands. The first group was made up of 89 male adolescents who were serving judicial sanctions in the juvenile justice system, ranging in age from 14 to 22 years (M = 17.42; SD = 1.53). Sentences as minors are imposed in Spain from 14 to 17 years of age, although it is possible to be serving a judicial measure up to 22 years of age. In this group, 68.5% (*n* = 62) admitted to having exercised CPV, although only 42.22% (*n* = 37) of these youths were serving judicial sanctions for this crime at the time of the study. Moreover, 42.22% of participants were serving judicial sanctions imposed for other causes (*n* = 37); the most frequent crimes were violent robbery (41.2%, *n* = 15), and nonviolent robbery (27.5%, *n* = 10). Crimes of assault or breaking and entering were each committed by three young persons (5.9%, *n* = 3); crimes of intimate partner violence and road safety violation by two each (3.9%, *n* = 4); and crimes of assault on authority, drug trafficking, crimes against sexual freedom, and attempted homicide by one person in each case (2%, *n* = 4). There was also one case (2%) of serving a second judicial sanction due to having violated a previous sanction for robbery. In this first group, 72.6% (*n* = 65) had a previous record.

The second group was made up of 70 men who were serving a prison sentence in the adult justice system, their ages ranging from 21 to 59 (M = 36.17; SD = 9.51). Of these inmates, 45.7% (*n* = 32) reported having exercised CPV in adolescence, although none of them were serving time for this reason at the time of the study. The crimes that had been committed were violent robbery (24.3%, *n* = 17), drug trafficking (12.9%, *n* = 9), nonviolent robbery (10%, *n* = 7), breach of sentence (10%, *n* = 7), domestic violence (8.6%, *n* = 6), and assault and battery (5.7%, *n* = 4). The crimes of resisting arrest and sexual coercion were committed by two participants each (2.9%, *n* = 4), while fraud, computer crime, and insults/threats were each committed by one person (1.4%, *n* = 3). The crime committed by 13 participants (18.5%) was missing. In this group, 62.1% (*n* = 43) had a previous record.

### 2.3. Instruments

The questionnaire used in this study included the following scales:

Self-Reported Child-to-Parent Violence: CPV was registered, as in Hernández [80], through the following question: “When living together with your parents or guardians, how often do you or did you do any of the following behaviors?” Participants answered 9 items which were selected based on Cottrell’s (2001) [81] definition, referring to behaviors intended to control and/or cause harm to parents, whether physical, psychological, emotional, or economic. These behaviors were: Insulting; running away from home; spitting; making obscene gestures; stealing; destroying their things; putting parents in debt; intimidating, blackmailing, or threatening them; hitting, punching, throwing objects at them, or pushing them. Participants were asked to answer on an 11-point Likert scale, from 0 (Never) to 10 (Very often). A total index of self-reported CPV was obtained by taking the average score of these 9 behaviors. Hernández [80] and Hernández et al. [64] have provided validity and reliability evidence for this instrument. In this study, the internal consistency values were: α = 0.85; CI95% [0.70–0.87]; ω = 0.84.

Exposure to Violence Scale by Orue and Calvete [82]. This instrument measures previous exposure to violence; it contains 21 items, 9 of which refer to direct exposure as a victim and 12 to indirect exposure as a witness. In each case, the items refer to three types of violence (physical, verbal and threats) in four contexts (school, street, home, and television). Indirect exposure to violence includes Seeing violence in the classroom, Seeing violence in the streets, Seeing violence at home, and Seeing violence on television. Direct exposure to violence includes Experiencing violence in the classroom, Experiencing violence in the streets, and Experiencing violence at home. Participants were asked to respond to each item on an 11-point Likert scale, from 0 (Never) to 10 (Every day). Several research studies have contributed validity and reliability tests for this scale [82]. In this study, the internal consistency values for the different subscales were as follows: Seeing violence in the classroom: α = 0.80; CI95% [0.74–0.84]; ω = 0.76; Seeing violence in the streets: α = 0.80; CI95% [0.74–0.84], ω = 0.76; Seeing violence at home: α = 0.80; CI95% [0.74–0.84], ω = 0.76; Seeing violence on television: α = 0.80; CI95% [0.74–0.84], ω = 0.76; Experiencing violence in the classroom: α = 0.80; CI95% [0.74–0.84], ω = 0.76; Experiencing violence in the streets: α = 0.80; CI95% [0.74–0.84], ω = 0.76; Experiencing violence at home: α = 0.80; CI95% [0.74–0.84], ω = 0.76.

Self-Concept Scale, Form 5, by García and Musitu [83]. This scale contains 30 items that measure 5 dimensions of self-concept: social, emotional, family, academic, and physical, plus global self-concept. Participants were asked to answer on an 11-point Likert scale, from 0 (Totally disagree) to 10 (Totally agree). Several research studies have contributed validity and reliability evidence for this scale [83]. In this study, the internal consistency values for the different subscales were as follows: academic self-concept: α = 0.82; CI95% [0.76–0.86], ω = 0.79; social self-concept: α = 0.82; CI95% [0.76–0.86], ω = 0.79; emotional self-concept: α = 0.82; CI95% [0.76–0.86], ω = 0.79; family self-concept: α = 0.82; CI95% [0.76–0.86], ω = 0.79; physical self-concept: α = 0.82; CI95% [0.76–0.86], ω = 0.79.

Interpersonal Reactivity Index by Davis [84], adapted to Spanish by Pérez-Albéniz et al. [85]. This scale measures different dimensions of Empathy and contains 28 items grouped into four variables: 1. Fantasy, reflecting the participant’s tendency to identify with fictitious characters in books and films; 2. Perspective taking, referring to participants’ tendency to adopt the perspective or viewpoint of other persons; 3. Empathic concern, which is related to the tendency to have feelings of compassion and concern for others; 4. Personal distress, which involves feelings of discomfort and anxiety that people experience when they witness the negative experiences of others. Participants were to indicate the degree to which the statements presented to describe how they usually think, feel or act, on an 11-point Likert scale from 0 (does not describe me at all) to 10 (describes me very well). Several research studies have contributed validity and reliability evidence for this scale [85]. In this study, the internal consistency values for the subscales were as follows: Fantasy: α = 0.83, CI95% [0.79–087], ω = 0.84; Perspective taking: α = 0.83, CI (95%) [0.79–087], ω = 0.84; Empathic concern: α = 0.83, CI95% [0.79–087], ω = 0.84; and Personal distress: α = 0.83, CI95% [0.79–087], ω = 0.84.

Social Problem-Solving Inventory-Revised by D’Zurilla et al. [86], adapted to Spanish by Maydeu-Olivares et al. [87]. This scale measures interpersonal problem-solving styles and problem orientation; the short version was used. Participants were to indicate the degree to which the 25 statements actually described the way they usually think, feel, or act, in general, when faced with important problems in their daily life. Participants answered on a five-point Likert-type scale from 0 (Not true of me at all) to 4 (Totally true). The scores were averaged for 2 dimensions of problem orientation and for 3 problem-solving styles. The dimensions of problem orientation are positive orientation and negative orientation. The problem-solving styles are rational style, impulsive style, and avoidant style. Positive orientation is a constructive attitude that implies a general disposition to consider problems as challenges and not threats, to believe in one’s own ability to solve problems, that problems are solvable, that a good solution to a problem takes time, effort, and persistence, and a commitment to quickly solve problems instead of avoiding them. A negative outlook is an attitude that implies a general tendency to see problems as threats, as unsolvable, to doubt one’s own ability to solve them, and to feel frustrated and disturbed when encountering problems in life. The rational style consists of deliberately, systematically applying problem-solving principles and techniques. The impulsive style implies consideration of few alternatives, going ahead with the first idea that comes up; superficial, hasty and nonreflexive exploration of alternatives and consequences, and a careless assessment of the results obtained. Finally, the avoidant style leads to avoiding problems instead of confronting them, putting off finding the solution, hoping that they solve themselves, or trying to get others to solve them. Several research studies have contributed validity and reliability evidence for this scale [88]. In this study, the internal consistency values were as follows: positive outlook: α = 0.80; CI95% [0.75–0.85], ω = 0.75 and negative outlook: α = 0.80; CI95% [0.75–0.85], ω = 0.75. In the case of problem-solving styles, for rational style it was α = 0.80; CI95% [0.75–0.85], ω = 0.75; impulsive style: α = 0.80; CI95% [0.75–0.85], ω = 0.75; and for avoidant style: α = 0.80; CI95% [0.75–0.85], ω = 0.75.

### 2.4. Procedure

First, permission to carry out the investigation was requested from the competent governmental authority, and the project was presented to those responsible for implementing the participants’ judicial sanction or sentence. According to the current law in the study setting, the governmental authority and those responsible for the judicial sanction/sentence are different for adolescents and for adults. Next, we asked the staff at the centers where the participants were located for instructions on the procedure to obtain their informed consent and, in the case of adolescents, consent from their legal guardians. By following their indications, we ensured that data collection would cause the least possible interference in the functioning of the center and in the participants’ daily activities.

In the participant interviews, they were informed of the project aims and reassured of the anonymity and confidentiality of the information they provided. Each participant answered the questionnaire individually or in small groups in the location where they were serving their judicial sanction or internment sentence, or in the case of adolescents who were already on probation, at the probation facilities. The questionnaire was administered as a semi-structured interview when the participant’s reading comprehension was low, regardless of whether it was an adolescent or an adult.

### 2.5. Data Analysis

In order to analyze group differences in the exposure-to-violence variables relative to the first two subobjectives, we conducted t-tests for independent samples, with Bonferroni adjustment to control the type I error rate, given the high number of comparisons to be made. A bootstrap procedure was used to correct the effect of noncompliance with distribution assumptions, when applicable. Effect sizes were estimated using Cohen’s d, with CI95%. Automatic regression models were used to estimate a self-reported CPV predictive model based on the dimensions of the psychological variables selected, comparing the estimate with optimal Bayesian methods to ensure the stability of the model under consideration. In this case, automatic regression models analyze all possible combinations of predictors using Akaike’s Criterion Information Corrected (AICC) and the forward stepwise procedure for the model selection method. The analyses were conducted with the statistical software SPSS 27 and JASP 0.14.1.

## 3. Results

**Figure 1:**
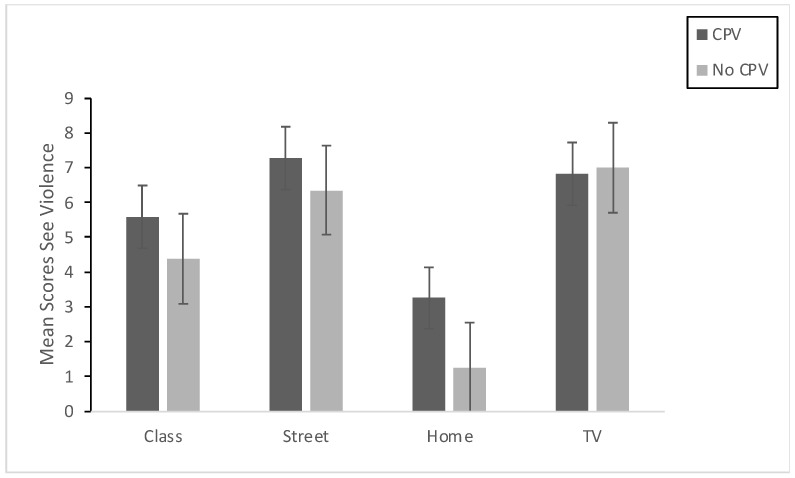
Means and Std. Error for indirect exposure to violence (Seeing violence in class, on the street, at home, and on TV) of the group that reported having committed CPV (CPV) and of the group that reported not having done so (No CPV).

**Figure 2:**
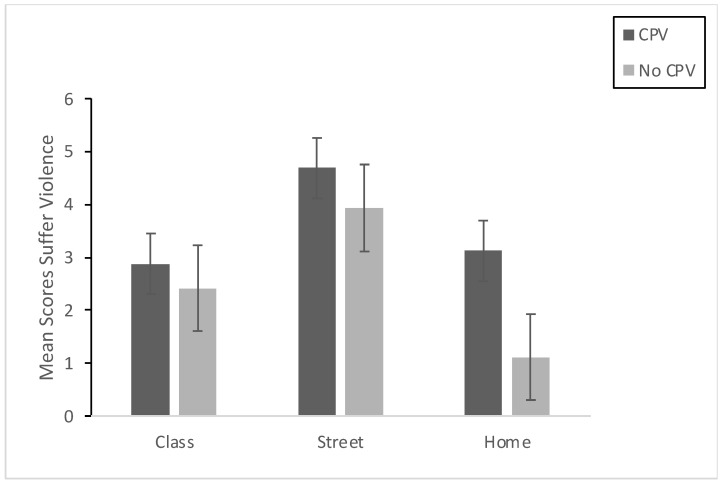
Means and Std. Error for direct exposure to violence (Experiencing violence in class, on the street, and at home) of the group that reported having committed CPV (CPV) and of the group that reported not having done so (No CPV).

First, we checked for between-group differences in the variables related to exposure to violence (seeing and experiencing violence), comparing the adolescents and the adults. We used *t*-tests for independent samples, with the Bonferroni correction and the per-comparison alpha set at 0.01. The estimation was also carried out by bootstrapping due to a lack of normality in some of the exposure-to-violence variables. The results indicated no statistically significant differences between adolescents and adults in any of the variables relating to seeing and experiencing violence (*p* > 0.01). This common pattern indicates that the two groups may be considered equivalent with regard to exposure to violence, whether experienced directly or indirectly. Taking into account this common pattern, we explored for possible statistically significant differences in the exposure-to-violence variables (seeing and experiencing violence) in the global sample as a function of whether or not CPV had been exercised. Participants were assigned to the CPV group when their score on the CPV scale was >1. The t-tests (adjusting the per-comparison alpha to 0.01 by means of Bonferroni correction and with bootstrapping) showed statistically significant differences between the two groups (see Table 1) in the following variables of indirect exposure to violence: Seeing violence in class (*p* < 0.01, d = 0.482; CI95% [−0.804, −0.158]) and at home (*p* < 0.01; d = 0.823; CI95% [−1.153, −0.490]) but not in Seeing violence on the street or on television (*p* > 0.01). Regarding the variables related to experiencing violence, the results showed statistically significant differences in Experiencing violence at home (*p* < 0.01, d = 0.902; CI95% [−1.235, −0.567]), but not in class or on the street (*p* > 0.01). Figure 1 and Figure 2 present the results graphically.

The predictive model for self-reported CPV was estimated using the psychological variables empathy, self-concept, problem orientation and problem-solving styles as predictive variables. The group variable adults/adolescents was also included in the equation to estimate any possible effect.

The best predictive model of CPV includes some of the dimensions of self-concept, specifically academic (β = −0.149) and family self-concept (β = −0.177), as well as the avoidant (β = 0.313) and rational problem solving (β = −0.239) styles, and the negative orientation toward problems (β = −0.533). Specifically, the predictive model indicates that the participants who acknowledged having exercised CPV have a more negative orientation towards problems, a more avoidant and a less rational problem-solving style, as well as lower family and academic self-concepts. The goodness of fit indices obtained with the classical model (*R*^2^_adj_ = 0.255) and with the Bayesian replica (*R*^2^ = 0.294; BF_M_ = 83.736; BF_10_ = 202.011) are adequate. None of the dimensions of empathy, nor the condition of adult vs. adolescent, had a statistically significant effect on self-reported.

## 4. Discussion

The main objective of this investigation was to establish the psychosocial profile of adolescents and adults who acknowledged having exercised CPV. For this purpose, we explored whether their possible exposure to different forms of violence, whether direct or indirect, was a common or differentiating factor, both within the group and when compared to those who did not acknowledge having exercised. We also analyzed whether the psychological variables of empathy, self-concept, problem orientation, and problem-solving styles could be significant predictors of self-reported.

The results have shown the existence of a CPV offender profile that is common to adolescents and adults. This result is especially important because studies of CPV, with few exceptions, have focused on minor-age youths. This is true even though the extreme form of CPV, parricide, is carried out by adults in 75% to 91% of cases [76]. If there is an escalation of violence in the continued abuse of offspring to parents, similar to that of other forms of abuse, where more serious behaviors are preceded and/or accompanied by milder and less censured expressions [77], CPV would not be expected to disappear in adulthood. This does not mean that all adolescents who abuse their parents as minors continue to do so as adults. As in other forms of antisocial behavior, CPV is likely to disappear in most cases when the neuropsychological maturation process is complete [89]. However, there will be a minority of offenders who continue to abuse their parents over their lifetime, in line with Moffitt’s distinction [90] between delinquents whose antisocial activity is limited to adolescence and those who persist throughout their lifetime. To the extent that we can identify the profile of these offenders at an early age, we may be able to intervene more intensively, preventing the more serious crimes where parents are victimized. The results of this study, although preliminary in nature, may be useful in this direction.

At this point, it must be noted that we decided to measure CPV by self-report, in contrast to other types of criminological measures, for several reasons. All participants had received a sentence and had files at the prison or at the entity in charge for supervising their judicial sanction. These files include the crime for which they have been convicted. However, the type of crime where CPV is included is addressed in articles 153.2 and 173.2 of the Penal Code, referring to domestic violence in general. For this reason, it is difficult to know for certain which member of the family unit had been the victim. Moreover, the crime for which they were serving a sentence or judicial sanction at the time may not have been CPV; they might have had a prior conviction for CPV or have exercised CPV without ever being convicted on that account. In fact, 68.5% of adolescents under judicial sanctions admitted having practiced CPV, although only 42.22% of those who were serving judicial sanctions did so for CPV at the time of the study. In the case of adults, the difference was even greater since 45.7% admitted to having practiced CPV, but only 8.6% had been convicted of domestic violence. In the case of adolescents, it was possible to establish that the sanctions for crimes of domestic violence referred to CPV, thanks to the collaboration of the prosecution team, but not so in the case of the prisoners since the adult criminal system and any related information are managed by the central government.

It is true that self-report measures can be questionable in forensic contexts since people involved with the judicial system, such as adolescents with judicial sanctions or adult prisoners, may hide negative characteristics and/or simulate positive characteristics they do not actually possess [91,92]. However, the participants were aware that their answers would not have any consequence in their sanction or sentence; they knew from the start that they were volunteers in a university study conducted by people outside the prison staff or the juvenile judicial system. Moreover, their responses were anonymous, and they were not asked about crimes committed or their behavior during their sentence/sanction but about behaviors referring to their relationship with their parents. For these reasons, it is reasonable to think that under the circumstances, any distortion in their answers would be minimal or nonexistent.

Regarding the psychosocial profile of CPV offenders that emerges from these results, once again the variables of exposure to domestic violence, as an observer and especially as a victim, are what best define the more specific profile of the CPV perpetrator, when compared to that of other offenders [20,26]. This relationship is also confirmed in the case of adults, who do not differ from the adolescents in any of the variables relating to exposure to violence. The effect size of exposure to violence in class is half that of the other two variables. In the study by [64], experiencing violence in the home was the variable with the most weight in the discriminating function that best differentiated adolescents convicted of CPV from adolescents convicted of other crimes, or from normal adolescents. Exposure to violence is a factor outside the individual, a stimulus that influences behavior, but not necessarily as a direct influence, as is suggested by the bi-directionality of violence hypothesis [27,42] and the theory of intergenerational transmission of violence [46]. Certain authors have expressed that exposure to violence promotes the belief that the use of violence is justified as a way to solve conflicts of any type [17,33,34,35,36,37]. The mediating role of social attitudes, values, emotional intelligence, and other indicators of social information processing is also suggested, with results that are still very disparate [19,29,55,93,94,95]. It is possible that exposure to violence acts on CPV directly, as a descriptive social norm, and indirectly, through the prescriptive social norm and the personal norm, simultaneously, as occurs with other types of behaviors, both the prosocial and the antisocial [96]. Future research should look deeper into this aspect.

Family self-concept also plays an important role in establishing the psychosocial profile of CPV offenders, appearing as a protective factor as opposed to the risk factors. This result is consistent with the study by Hernández et al. [64], where self-concept was one of the variables that characterized adolescents under judicial sanctions for CPV, as stated above, in comparison to other delinquent and nondelinquent adolescents. This consistency is logical since family self-concept is the dimension of self-concept that most closely relates to intrafamily violence, not only because it refers to the same domain but because family relationships play an important role in the origin, maintenance, and desistance from offending behavior [96,97]. In this study, academic self-concept also differentiated between those who acknowledged having practiced CPV and those who did not, in the same line as studies that relate both family self-concept and academic self-concept to cyber-victimization [98] and to revenge motivation [99] in non-delinquent adolescents. Both family self-concept and academic self-concept play a role as protective factors; as these variables increase, CPV decreases. These results are also useful to show that analysis of the relationship between CPV and self-concept successfully addresses the issue of inconsistent results in relation to self-esteem.

None of the variables referring to empathy (fantasy, perspective taking, empathic concern, personal distress) were related to CPV. This result concurs with studies claiming that, while low empathy is linked to antisocial behavior in general [50,51], its relation to delinquent behavior is moderate and is limited to the cognitive dimension [52]. A relationship with CPV has been found only in the empathy attributed by supervisors of judicial sanctions to adolescents sentenced for this kind of crime when compared to those sentenced for other crimes [48,49,53]. It is, therefore, unclear if their empathy really is lower or if the expectations of these supervisors are influencing their assessment. In the study by Castañeda et al. [47], self-report measures were used, but they refer to the MACI Social Insensitivity scale, related to but not exactly equivalent to empathy. Even so, the difference was much less than differences found in expressed concerns about abuses in childhood and family discord. In the study by Contreras et al. [54], empathy was not related to any of the elements of the CPV model that the authors tested.

The results from this study related CPV to family and academic self-concept, but the psychological variables with the highest regression coefficients were negative orientation toward problems, followed by an Avoidant problem-solving style. The rational style was a protective factor, but its relationship to CPV was weaker than that of the preceding variables. These results are consistent with those of Contreras and Cano [60], who found that adolescents with judicial sanctions for CPV were deficient in a number of important capacities in solving interpersonal problems: field independence; noticing and retaining relevant information about social situations; anticipating and understanding the consequences of social behaviors; and selecting the appropriate means to achieve one’s goals. These deficits coexisted, logically, with higher impulsivity. These results are also consistent with studies where a relationship appears between executive functioning and antisocial behavior in general [100] and CPV in particular [101]. This is so in that executive functioning is closely tied to the attention, planning, and cognitive flexibility that are required for an adaptive solution of interpersonal problems.

This study presents certain limitations. First, it deals only with male offenders, so the conclusions should not be generalized to female samples until the results are replicated in such samples. Secondly, the study is cross-sectional and retrospective, so the relationships do not imply any kind of causality. We do not know whether the prisoners who acknowledged having practiced CPV continue to do so or not, nor if their responses might be influenced by processes of recall. Moreover, we do not know who among those who committed CPV as adolescents have never been incarcerated. Research in CPV requires longitudinal studies that extend into adulthood, not only across adolescence, in order to give a definite answer to the question posed by the Attorney General’s Office in its 2020 Annual Report [7], regarding what has become of those who battered parents when they were adolescents and who are now adults.

## 5. Conclusions

This study contributes to the development of CPV research by offering, for the first time, data on CPV in adults and data on how CPV is related to problem orientation and problem-solving styles. These results suggest that intervention programs with adolescents who batter their parents should incorporate interpersonal problem-solving skills so that when facing the inevitable parent-offspring conflicts, strategies are used that promote coexistence and not violence. In the same way that training in this type of skill significantly reduces recidivism, regardless of how the criminal behavior originated, it is possible that training CPV offenders in interpersonal problem-solving may help to control CPV behaviors [75]. As mentioned above, most cases of CPV are likely to disappear as its adolescent perpetrators complete their neuropsychological maturation process [89]. Nevertheless, regardless of how future longitudinal studies answer this question, training in interpersonal problem-solving skills could be helpful in reducing CPV in the present.

## Figures and Tables

**Table 1:** Bootstrap ^a^ for Independent Samples Test (DVs = seeing and experiencing violence; IV = CPV or not CPV).

		Mean Difference	Bias	Std. Error	Sig. (2-Tailed)	BCa 95% Confidence Interval	
						Lower	Upper
Seeing violence in class	Equal variances assumed	−1.35069	0.00738	0.43919	0.004	−2.17145	−0.49250
Seeing violence on the street	Equal variances not assumed	−0.99059	0.01512	0.41637	0.018	−1.81348	−0.09740
Seeing violence at home	Equal variances not assumed	−1.93678	0.00236	0.40667	0.001	−2.71059	−1.11803
Seeing violence on TV	Equal variances assumed	0.15958	−0.00073	0.46882	0.731	−0.79292	1.09108
Experiencing violence in class	Equal variances assumed	−0.54566	−0.01308	0.39566	0.164	−1.36621	0.22250
Experiencing violence on the street	Equal variances not assumed	−0.76760	−0.02093	0.39332	0.062	−1.54477	−0.04823
Experiencing violence at home	Equal variances not assumed	−2.12664	−0.00462	0.41073	0.001	−2.95129	−1.34653

^a^: Unless otherwise noted, bootstrap results are based on 1000 bootstrap samples.

## Data Availability

The raw data supporting the conclusions of this article will be made available by the authors without undue reservation.

## References

[1] Calvete E., Pereira R., Calvete E., Pereira R. (2019). Conceptualización de la Violencia Parental, Magnitud y Teorías. La Violencia Filio-Parental: Análisis, Evaluación e Intervención.

[2] Fiscalía General del Estado Memoria Annual 2004 [Attorney General’s Office, 2004 Annual Report]. Chrome-extension://efaidnbmnnnibpcajpcglclefindmkaj/viewer.html?pdfurl=https%3A%2F%2Fwww.fiscal.es%2Fdocuments%2F20142%2F9181b52e-6773-782d-5bbf-bf46c4d3878e.

[3] Pereira R. (2006). Violencia Filio-Parental: Un Fenómeno Emergente [Child-to-Parent Violence: An Emerging Phenomenon]. Rev. Mos..

[4] Fiscalía General del Estado Memoria Anual 2006 [Attorney General’s Office, 2006 Annual Report]. Chrome-extension://efaidnbmnnnibpcajpcglclefindmkaj/viewer.html?pdfurl=https%3A%2F%2Fwww.fiscal.es%2Fdocuments%2F20142%2F9181b52e-6773-782d-5bbf-bf46c4d3878e.

[5] Fiscalía General del Estado Memoria Anual 2007 [Attorney General’s Office, 2007 Annual Report]. Chrome-extension://efaidnbmnnnibpcajpcglclefindmkaj/viewer.html?pdfurl=https%3A%2F%2Fwww.fiscal.es%2Fdocuments%2F20142%2Fe8840872-5158-8285-a17a-76386fe76203.

[6] Fiscalía General del Estado Memoria Anual 2016 [Attorney General’s Office, 2016 Annual Report]. https://www.fiscal.es/memorias/memoria2016/FISCALIA_SITE/index.html.

[7] Fiscalía General del Estado Memoria Anual 2020 [Attorney General’s Office, 2020 Annual Report]. Annual Report. https://www.fiscal.es/memorias/memoria2020/FISCALIA_SITE/index.html.

[8] Fiscalía General del Estado Memoria Anual 2011 [Attorney General’s Office, 2011 Annual Report]. Chrome-extension://efaidnbmnnnibpcajpcglclefindmkaj/viewer.html?pdfurl=https%3A%2F%2Fwww.fiscal.es%2Fdocuments%2F20142%2F8e16bfcb-8140-3f23-2d72-ffd5d483f35e.

[9] Carrasco N., Garcia J., Zaldívar F., De Almería E.U., De Almería U. (2018). Diferencias asociadas a la violencia filio-parental en función del tipo de familia (“normalizadas” vs “en riesgo”) y parentesco de la víctima. Rev. Psic. Clín. Niñ. Adoles..

[10] Circular 1/2010, de 23 de Julio, Sobre el Tratamiento desde el Sistema de Justicia Juvenil de los Malos Tratos de Menores Contra sus Ascendientes [Circular 1/2010, 23 July 2010, on the Treatment from the Juvenile Justice System of Abuse by Minors against their Ascendants]. https://www.boe.es/buscar/doc.php?coleccion=fiscalia&id=FIS-C-2010-00001.

[11] Garrido V. (2005). Los Hijos Tiranos: El Síndrome del Emperador [The Tyrant Sons: The Emperor’s Syndrome].

[12] Pereira R., Bertino L. (2009). Una comprensión ecológica de la violencia filio-parental [An ecological understanding of child to parent violence]. Redes.

[13] Urra J. (2006). El Pequeño Dictador. Cuando los Padres Son las Víctimas [The Little Dictator. When Parents Are the Victims].

[14] Del Hoyo-Bilbao J., Orue I., Gámez-Guadix M., Calvete E. (2019). Multivariate Models of Child-to-Mother Violence and Child-to-Father Violence among Adolescents. Eur. J. Psychol. Appl. Leg. Context.

[15] Loinaz I., De Sousa A.M. (2019). Assessing Risk and Protective Factors in Clinical and Judicial Child-to-Parent Violence Cases. Eur. J. Psychol. Appl. Leg. Context.

[16] Beckmann L., Bergmann M.C., Fischer F., Mößle T. (2017). Risk and Protective Factors of Child-to-Parent Violence: A Comparison Between Physical and Verbal Aggression. J. Interpers. Violence.

[17] Calvete E., Orue I., Sampedro R. (2011). Violencia filio-parental en la adolescencia: Características ambientales y personales. Infancia Aprendiz..

[18] Jardim D.L., Groves E.S., Breitfeld P.P., Kurzrock R. (2017). Factors associated with failure of oncology drugs in late-stage clinical development: A systematic review. Cancer Treat. Rev..

[19] Contreras L., Cano Lozano M.d.C. (2016). Child-to-parent violence: The role of exposure to violence and its relationship to social-cognitive processing. Eur. J. Psychol. Appl. Leg. Context.

[20] Gallego R., Novo M., Fariña F., Arce R. (2019). Child-to-parent Violence and Parent-to-child Violence: A Meta-analytic Review. Eur. J. Psychol. Appl. Leg. Context.

[21] Ibabe I., Bentler P.M. (2015). The Contribution of Family Relationships to Child-to-Parent Violence. J. Fam. Violence.

[22] Ibabe I., Jaureguizar J., Bentler P.M. (2013). Risk Factors for Child-to-Parent Violence. J. Fam. Violence.

[23] Margolin G., Baucom B.R. (2014). Adolescents’ Aggression to Parents: Longitudinal Links with Parents’ Physical Aggression. J. Adolesc. Health.

[24] Izaguirre A., Calvete E. (2016). Exposure to Family Violence as a Predictor of Dating Violence and Child-to-Parent Aggression in Spanish Adolescents. Youth Soc..

[25] Perkins S.C., Smith-Darden J., Ametrano R.M., Graham-Bermann S.A. (2014). Typologies of Violence Exposure and Cognitive Processing in Incarcerated Male Adolescents. J. Fam. Violence.

[26] Simmons M., McEwan T.E., Purcell R., Ogloff J. (2018). Sixty years of child-to-parent abuse research: What we know and where to go. Aggress. Violent Behav..

[27] Ulman A., Straus M.A. (2003). Violence by Children Against Mothers in Relation to Violence Between Parents and Corporal Punishment by Parents. J. Comp. Fam. Stud..

[28] Kitzmann K.M., Gaylord N.K., Holt A.R., Kenny E.D. (2003). Child witnesses to domestic violence: A meta-analytic review. J. Consult. Clin. Psychol..

[29] Calvete E., Orue I., Gamez-Guadix M., Bushman B.J. (2015). Predictors of child-to-parent aggression: A 3-year longitudinal study. Dev. Psychol..

[30] Calvete E., Orue I., Fernández-González L., Chang R., Little T.D. (2019). Longitudinal Trajectories of Child-to-Parent Violence through Adolescence. J. Fam. Violence.

[31] Paterson R., Luntz H., Perlesz A., Cotton S. (2002). Adolescent Violence towards Parents: Maintaining Family Connections When the Going Gets Tough. Aust. New Zealand J. Fam. Ther. (ANZJFT).

[32] Moffitt T., Caspi A., Farrington D., Coid J. (2003). Preventing the inter-generational continuity of antisocial behaviour: Implications of partner violence. Early Prevention of Adult Antisocial Behavior.

[33] García A.L.C., Molina E.F., Alberola C.R. (2008). Menores agresores en el hogar [Child molesters in the home]. Bol. Crim..

[34] Ibabe I. (2007). Perfil de los Hijos Adolescentes que Agreden a Sus Padres. Investigación Realizada en la Comunidad Autónoma Vasca [Profile of Adolescent Children Who Assault Their Parents. Research Carried out in the Basque Autonomous Community].

[35] Ibabe I., Jaureguizar J. (2011). ¿Hasta qué punto la violencia filio-parental es bidireccional?. An. Psicol..

[36] Rechea C., Cuervo A. (2010). Menores Agresores en el Ámbito Familiar [Aggressive Minors in the Family Environment].

[37] Suárez B. (2012). Violencia filio-parental: Aproximación a un fenómeno emergente [Chil-to-parent violence: An approach to an emerging phenomenon]. Rev. Humanid. Cienc. Soc..

[38] Ibabe I. (2020). A Systematic Review of Youth-to-Parent Aggression: Conceptualization, Typologies, and Instruments. Front. Psychol..

[39] Laporte L., Jiang D., Pepler D.J., Chamberland C. (2009). The Relationship Between Adolescents’ Experience of Family Violence and Dating Violence. Youth Soc..

[40] Fernández-González L., Orue I., Adrián L., Calvete E. (2021). Child-to-Parent Aggression and Dating Violence: Longitudinal Associations and the Predictive Role of Early Maladaptive Schemas. J. Fam. Violence.

[41] Brezina T. (1999). Teenage violence towards parents as an adaptation to family strain: Evidence from a National survey of male adolescents. Youth Soc..

[42] González M., Morán N., Redondo N., García M., Urra J. (2015). Análisis de Reciprocidad de la violencia en la violencia filio-parental [Analysis of reciprocity of violence in child-to-parent violence]. I Congreso Nacional de Violencia Filio-Parental.

[43] Kennedy T.D., Edmonds W.A., Dann K.T.J., Burnett K.F. (2010). The Clinical and Adaptive Features of Young Offenders with Histories of Child-Parent Violence. J. Fam. Violence.

[44] Routt G., Anderson L. (2011). Adolescent Violence towards Parents. J. Aggress. Maltreat. Trauma.

[45] Black D.S., Sussman S., Unger J. (2009). A Further Look at the Intergenerational Transmission of Violence: Witnessing Interparental Violence in Emerging Adulthood. J. Interpers. Violence.

[46] Haselschwerdt M.L., Savasuk-Luxton R., Hlavaty K. (2017). A Methodological Review and Critique of the “Intergenerational Transmission of Violence” Literature. Trauma Violence Abus..

[47] Castañeda A., Garrido-Fernández M., Lanzarote M.-D. (2012). Menores con conducta de maltrato hacia los progenitores: Un estudio de personalidad y estilos de socialización [Minors with abusive behavior towards parents: A study on personality and socialization styles]. Rev. Psicol. Soc..

[48] Ibabe I., Jaureguizar J. (2010). Child-to-parent violence: Profile of abusive adolescents and their families. J. Crim. Justice.

[49] Ibabe I., Jaureguizar J., Diaz O. (2009). Adolescent violence against parents: Is it a consequence of gender inequality?. Eur. J. Psychol. Appl. Leg. Context.

[50] Lovett B.J., Sheffield R.A. (2007). Affective empathy deficits in aggressive children and adolescents: A critical review. Clin. Psychol. Rev..

[51] Schaffer M., Clark S., Jeglic E.L. (2008). The Role of Empathy and Parenting Style in the Development of Antisocial Behaviors. Crime Delinq..

[52] Férriz L., Sobral J., Fraguela X.A.G. (2017). Empatía y delincuencia juvenil: Un meta-análisis sobre la relación [Empathy and juvenile delinquency: A meta-analysis of the relationship]. Rev. Iber. Psic. Sal..

[53] Ibabe I., Jaureguizar J., Díaz O. (2007). Violencia Filio-Parental. Conductas Violentas de Jóvenes Hacia sus Padres [Child-to-Parent Violence. Violent Behaviors of Young People towards Their Parents].

[54] Contreras L., León S.P., Cano-Lozano M.C. (2020). Socio-cognitive variables involved in the relationship between violence exposure at home and child-to-parent violence. J. Adolesc..

[55] Donnellan M.B., Trzesniewski K.H., Robins R.W., Moffitt T.E., Caspi A. (2005). Low Self-Esteem Is Related to Aggression, Antisocial Behavior, and Delinquency. Psychol. Sci..

[56] Calvete E., Gámez-Guadix M., Orue I. (2014). Características familiares asociadas a violencia filio-parental en adolescentes [Family characteristics associated with child- to-parent violence in adolescents]. An. Psicol..

[57] Elliott R., Bohart A.C., Watson J.C., Greenberg L.S. (2011). Empathy. Psychotherapy.

[58] Ibabe I. (2014). Direct and indirect effects of family violence on child-to-parent violence. Stud. Psychol..

[59] Ibabe I., Jaureguizar J. (2011). El perfil psicológico de los menores denunciados por violencia filio-parental [The psychological profile of minors reported for child-to-parent violence]. Rev. Esp. Inv. Crim..

[60] Contreras L., Cano M.C. (2014). Exploring psychological features in adolescents who assault their parents: A different profile of young offenders?. J. Forensic Psychiatry Psychol..

[61] Ibabe I., Arnoso A., Elgorriaga E. (2014). Behavioral problems and depressive symptomatology as predictors of child-to-parent violence. Eur. J. Psychol. Appl. Leg. Context.

[62] Ibabe I., Arnoso A., Elgorriaga E. (2014). The clinical profile of adolescent offenders of child-to-parent violence. Procedia Soc. Behav. Sci..

[63] Vilariño M., Arce R., Fariña F. (2013). Forensic-clinical interview: Reliability and validity for the evaluation of psychological injury. Eur. J. Psychol. Appl. Leg..

[64] Hernández A., Martín A.M., Hess-Medler S., García-García J. (2020). What Goes on in This House Do Not Stay in This House: Family Variables Related to Adolescent-to-Parent Offenses. Front. Psychol..

[65] D’Zurilla T.J., Nezu A.M. (2007). Problem-Solving Therapy: A Positive Approach to Clinical Intervention.

[66] Nock M.K., Kazdin A.E. (2002). Examination of Affective, Cognitive, and Behavioral Factors and Suicide-Related Outcomes in Children and Young Adolescents. J. Clin. Child Adolesc. Psychol..

[67] Mcmurran M., Duggan C., Christopher G., Huband N. (2007). The relationships between personality disorders and social problem solving in adults. Pers. Individ. Differ..

[68] Frick P.J. (1998). Conduct Disorders and Severe Antisocial Behavior.

[69] Keltikangas-Järvinen L., Pakaslahti L. (1999). Development of social problem-solving strategies and changes in aggressive behavior: A 7-year follow-up from childhood to late adolescence. Aggress. Behav..

[70] Herrick S.M., Elliot T.R. (2001). Social problem-solving abilities and personality disorder characteristics among dual-diagnosed persons in substance abuse treatment. J. Clin. Psychol..

[71] Lösel F., Bender D., Farrington D.P., Coid J. (2003). Protective factors and resilience. Early Prevention of Adult Antisocial Behavior.

[72] Matthys W., Lochman J., McMurran M., McGuire J. (2005). Social Problem Solving in Aggressive Children. Social Problem Solving and Offending.

[73] Mathys C. (2017). Effective components of interventions in juvenile justice facilities: How to take care of delinquent youths?. Child. Youth Serv. Rev..

[74] McGuire J., Hollin C.R., Palmer E.J. (2006). General offending behaviour programmes: Concept, theory, and practice. Offending Behaviour Programmes: Development, Application, and Controversies.

[75] Polaschek D., Day A., Hollin C. (2019). Correctional Psychology: A Short History and Current Standing. The Wiley International Handbook of Correctional Psychology.

[76] Heide K.M., Petee T.A. (2007). Parricide: An Empirical Analysis of 24 Years of U.S. Data. J. Interpers. Violence.

[77] Cortina H., Martín A.M. (2020). La especificidad conductual de la violencia filio-parental. An. Psicol..

[78] Holt A., Shon P.C. (2016). Exploring Fatal and Non-Fatal Violence Against Parents: Challenging the Orthodoxy of Abused Adolescent Perpetrators. Int. J. Offender Ther. Comp. Criminol..

[79] Oliva A., Ríos M., Antolín L., Parra A., Hernando A., Pertegal M. (2010). Más allá del déficit: Construyendo un modelo de desarrollo positivo adolescente. Infanc. Aprendiz..

[80] Hernández A. (2016). El Perfil Psicosocial de los Agresores y de las Víctimas de la Violencia Filioparental [Psychosocial Profile of Aggressors and Victims of Child-to-Parent Violence]. Ph.D. Thesis.

[81] Cottrell B. (2001). Parent Abuse: The Abuse of Parents by Their Teenage Children.

[82] Orue I., Calvete E. (2010). Elaboración y validación de un cuestionario para medir la exposición a la violencia en infancia y adolescencia [Development and validation of a questionnaire to measure exposure to violence in childhood and adolescence]. Rev. Int. Psicol. Ter. Psicol..

[83] García F., Musitu G. (2014). Autoconcepto Forma 5 [Sef-concept Form 5].

[84] Davis M.H. (1980). A multi-dimensional approach to individual differences in empathy. JSAS Catalog Selec. Doc. Psych..

[85] Pérez-Albéniz A., De Paúl J., Etxeberría J., Montes M.P., Torres E. (2003). Adaptación de Interpersonal Reactivity Index (IRI) al español [Adaptation of the Interpersonal Reactivity Index (IRI) to Spanish]. Psicothema.

[86] D’Zurilla T., Nezu A., Maydeu-Olivares T. (1999). Social Problem-Solving Inventory Revised (SPSI-R): Manual.

[87] Maydeuolivares A., Rodriguezfornells A., Gomezbenito J., Dzurilla T. (2000). Psychometric properties of the Spanish adaptation of the Social Problem-Solving Inventory-Revised (SPSI-R). Pers. Individ. Differ..

[88] Aburezeq K., Kasik L. (2021). The social problem solving inventory–revised as a measurement of individuals’ social problems solving: Review of modern literature. Rom. J. Exp. Appl. Psychol..

[89] van Goozen S.H., Langley K., Hobson C.W. (2021). Childhood Antisocial Behavior: A Neurodevelopmental Problem. Annu. Rev. Psychol..

[90] Moffitt T.E. (1993). Adolescence-limited and life-course-persistent antisocial behavior: A developmental taxonomy. Psychol. Rev..

[91] Arce R., Fariña F., Vilariño M. (2015). Daño psicológico en casos de víctimas de violencia de género: Estudio comparativo de las evaluaciones forenses. Rev. Iberoam. Psicol. Salud.

[92] Fariña F., Redondo L., Seijo D., Novo M., Arce R. (2017). A meta-analytic review of the MMPI validity scales and indexes to detect defensiveness in custody evaluations. Int. J. Clin. Health Psychol..

[93] Contreras L., Cano M.C. (2016). Social Competence and Child-to-Parent Violence: Analyzing the Role of the Emotional Intelligence, Social Attitudes, and Personal Values. Deviant Behav..

[94] Orue I., Calvete E., Fernández-González L. (2019). Early Maladaptive Schemas and Social Information Processing in Child-to-Parent Aggression. J. Interpers. Violence.

[95] Martín A.M., Hernández B., Hess S., Frías-Armenta M. (2012). Why ordinary people comply with environmental laws: A structural model on normative and attitudinal determinants of illegal anti-ecological behaviour. Leg. Criminol. Psychol..

[96] Martín A.M., Padrón F., Redondo S. (2019). Early Narratives of Desistance from Crime in Different Prison Regimes. Eur. J. Psychol. Appl. Leg. Context.

[97] Redondo S., Padrón-Goya F., Martín A.M. (2021). Offenders’ Narratives on Criminal Desistance While Serving a Prison Sentence. Vict. Offenders.

[98] Romero-Abrio A., León-Moreno C., Musitu-Ferrer D., Villarreal-González M.E. (2019). Family Functioning, Self-Concept and Cybervictimization: An Analysis Based on Gender. Soc. Sci..

[99] León-Moreno C., Musitu-Ferrer D. (2019). Estilos de comunicación familiar, autoconcepto escolar y familiar, y motivación de venganza en adolescentes. Eur. J. Investig. Health Psychol. Educ..

[100] Gil-Fenoy M.J., García-García J., Carmona-Samper E., Ortega-Campos E. (2018). Antisocial Behavior and Executive Functions in Young Offenders. Rev. Psicodidáctica.

[101] Fandiño R., Basanta J., Sanmarco J., Arce R., Fariña F. (2021). Evaluation of the Executive Functioning and Psychological Adjustment of Child-to-Parent Offenders: Epidemiology and Quantification of Harm. Front. Psychol..

